# Supplementary material for: Virus lasers for biological detection
Source: Nat Commun. 2019 Aug 9;10:3594. doi: 10.1038/s41467-019-11604-z (PMC6689004; doi:10.1038/s41467-019-11604-z)
Supplement: Supplementary file 1 — Supplementary Information [file 41467_2019_11604_MOESM1_ESM.pdf]

Virus lasers for biological detection

Supplementary information

John E. Hales[1]*, Guy Matmon[2], Paul A. Dalby[1], John M. Ward[1] & Gabriel Aeppli[2-4]*

[1]Department of Biochemical Engineering, University College London, Bernard Katz Building, Gower Street, London, WC1E 6BT.

[2]Paul Scherrer Institut, Villigen PSI CH-5232, Switzerland.

[3]Department of Physics, ETH Zürich, Zürich CH-8093, Switzerland.

[4]Institut de Physique, EPFL, Lausanne CH-1015, Switzerland.

*Correspondence to: john.hales@ucl.ac.uk (J.E.H.); gabriel.aeppli@psi.ch (G.A.)

Supplementary Discussion

Extended technical description of proof-of-concept mix-and-measure ligand-binding assay

Other experimental factors might have resulted in a decrease in the virus laser output intensity (Fig. 4), and we consider three possibilities here. The first and most trivial is the dilution of the dye with the addition of antibody solution. This is ruled out on arithmetic grounds: the 5 µm pore size of the filter was much larger than M13 and the addition of 100 µL buffer to the 6.85 mL reservoir would not have had a profound effect unless the initial concentration of probes was less than 1.5 % above the minimum required to achieve lasing. The second is air bubbles in the cuvette chamber. Whilst air did sometimes pass through the flow cuvette between measurements, the system was designed to allow air to escape and the variation of the intensity did not increase as the mean decreased, which would indicate a trapped bubble partially occluding the resonant cavity (Fig. 4). Third, because of the repetitive nature of the experiment, photobleaching effects could produce reductions in intensity which might be confused with those due to the binding of the antibody. For instance, photobleaching of the dyes most likely caused the decrease in the intensity below threshold (Supplementary Fig. 7), and the intensity above threshold between the spectral measurement, the threshold measurement and before adding cp-mAb in the time-series measurement (Fig. 4). However, photobleaching does not account for the step changes in the intensity during the time-series measurement (Fig. 4c, Supplementary Fig. 9): there was no decrease below threshold and there were only 295 excitation pulses in total compared to 1340 excitation pulses in total for the preceding spectral and threshold measurements. In further experiment 2, photobleaching did not cause step changes in the threshold point and the addition of cp-mAb led to a sharp increase in the threshold point (Fig. 5a, Supplementary Fig. 10). In further experiment 1, neither photobleaching nor the addition of a non-binding antibody resulted in step changes in the threshold point, and the subsequent addition of

cp-mAb resulted in a sharp increase in the threshold point after a short lag period (Fig. 5b, Supplementary Fig. 10). The effect of the addition of cp-mAb may have been more immediate in further experiment 2 than in further experiment 1 because more cp-mAb was added. The greater output intensity from the virus laser in further experiment 2 immediately prior to the addition of cp-mAb may also have been a contributory factor.

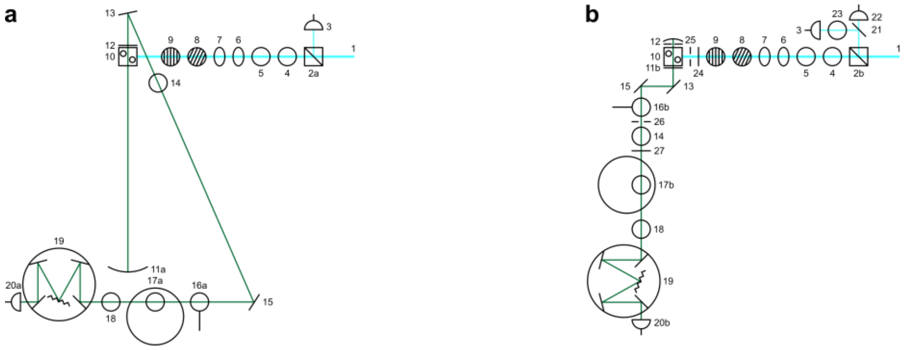

Supplementary Figure 1 | Optical configuration diagrams. Optical configuration for experiments conducted using, **a**, resonant cavity R1 and, **b**, resonant cavity R2. The numerical labels in **a** and **b** refer to the following list of components: 1. Optical parametric oscillator via periscope with two flat 1 " Ag mirrors. 2a. Pellicle beamsplitter. 2b. Beamsplitting window. 3. Beam monitor: pyroelectric probe connected to a Joulemeter. 4, 5. Spherical beam-expanding telescope. 6, 7. Cylindrical beam-reducing telescope. 8, 9. Pump energy control: two Glan-Laser calcite polarizers, 8 in a motorised rotation stage, 9 fixed in a manual rotation stage. 10. Flow cuvette with 40 μL chamber. 11a. Spherical dielectric mirror, $R = 400$ mm. 11b. 300 μm pinhole bonded to a spherical Ag mirror, $R = 50$ mm. 12. Flat, dielectric output coupler. 13. Flat 1 " Ag mirror. 14. 1 " spherical convex lens. 15. Flat 1 " Ag mirror. 16a. OD 4 reflective neutral density filter in manual flipper mount. 16b. OD 4 reflective neutral density filter in motorised flipper mount. 17a. Manual filter wheel mounted with OD 0.5, 1.0, 2.0, 3.0 reflective neutral density filters, and one unmounted position. 17b. Motorised filter wheel mounted with OD 0.5, 1.0, 2.0, 3.0, 4.0 reflective neutral density filters, and one unmounted position. 18. Spherical convex lens. 19. Monochromator with a 1200 grooves mm^{-1} grating. 20a. Detector: Photomultiplier tube connected to a 50 Ω terminator, voltage measured by a 60 MHz oscilloscope. 20b. Same as 20a, except with a 500 MHz oscilloscope and a 1 GHz feed-through 50 Ω terminator. 21. Beamsplitting flat 1 " Al mirror. 22. Optical trigger to oscilloscope: Si photodiode connected to a high-speed circuit preceded by an OD 2.0 reflective neutral density filter. 23. 1 "

spherical concave lens. 24. Shortpass filter, $\lambda = 510$ nm. 25. Rectangular aperture. 26. Iris diaphragm. 27. Longpass filter, $\lambda = 500$ nm.

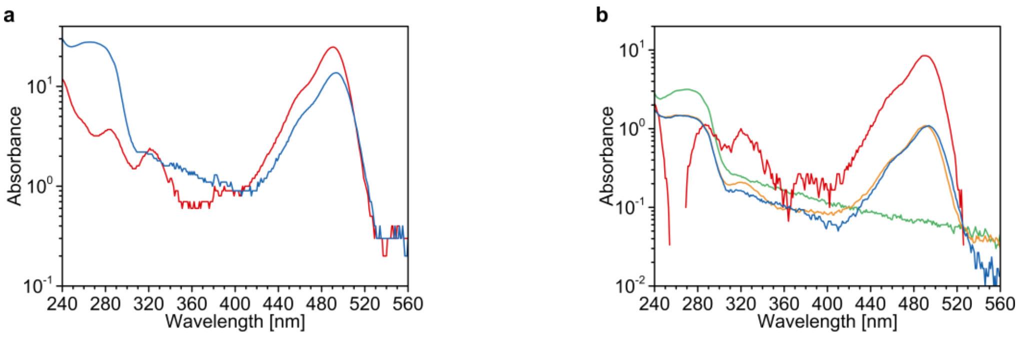

Supplementary Figure 2 | Absorption spectra. **a**, Absorption spectra of 413 pmol mL^{-1} M13 conjugated with 386 dyes per M13 (dark blue) and 301 nmol mL^{-1} fluorescein (red). **b**, Absorption spectra of 50 pmol mL^{-1} M13 (green), 102 nmol mL^{-1} fluorescein (red), 23 pmol mL^{-1} M13 conjugated with 564 dyes per M13 (dark blue) and 23 pmol mL^{-1} M13 mixed with 12.9 nmol mL^{-1} fluorescein (orange). Source data are provided as a Source Data file.

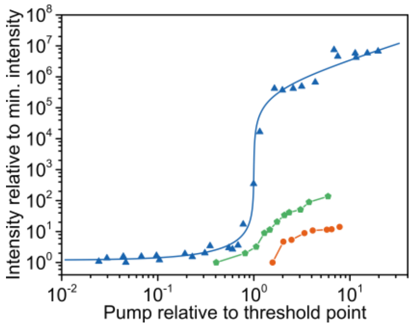

Supplementary Figure 3 | Comparison to threshold curves from literature. The threshold behaviour of 351 pmol mL^{-1} dye-labelled M13 (dark blue) (see Supplementary Fig. 6 for more examples) is compared to the measured response to pumping of a eGFP-expressing cell laser (green) (*7*, Fig. 2e) and a cascade optofluidic ring resonator FRET laser consisting of a DNA scaffold bridging Cy3, Cy5 and Cy5.5 dyes (orange) (*21*, Fig. 5b). These examples from the biological laser literature were selected due to their outstanding significance to the field. To enable comparison, the pump values have been divided by the reported threshold point and the intensity values have been divided by the lowest recorded non-zero intensity value.

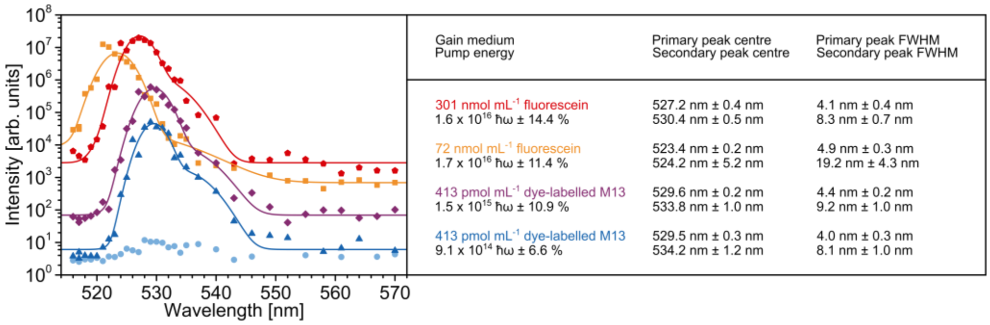

Gain medium<br>Pump energy	Primary peak centre<br>Secondary peak centre	Primary peak FWHM<br>Secondary peak FWHM
301 nmol mL^{-1} fluorescein<br>1.6×10^{16} ħω ± 14.4 %	527.2 nm ± 0.4 nm<br>530.4 nm ± 0.5 nm	4.1 nm ± 0.4 nm<br>8.3 nm ± 0.7 nm
72 nmol mL^{-1} fluorescein<br>1.7×10^{16} ħω ± 11.4 %	523.4 nm ± 0.2 nm<br>524.2 nm ± 5.2 nm	4.9 nm ± 0.3 nm<br>19.2 nm ± 4.3 nm
413 pmol mL^{-1} dye-labelled M13<br>1.5×10^{15} ħω ± 10.9 %	529.6 nm ± 0.2 nm<br>533.8 nm ± 1.0 nm	4.4 nm ± 0.2 nm<br>9.2 nm ± 1.0 nm
413 pmol mL^{-1} dye-labelled M13<br>9.1×10^{14} ħω ± 6.6 %	529.5 nm ± 0.3 nm<br>534.2 nm ± 1.2 nm	4.0 nm ± 0.3 nm<br>8.1 nm ± 1.0 nm

Supplementary Figure 4 | R1 emission spectra. The colours of the scatter points and fitted curves match the colours in the table of fit parameters, which also includes the mean and coefficient of variation of the pump energy. The errors in the peak centres and FWHMs are standard errors. A pump energy of 5.5×10^{14} photons $pulse^{-1}$ ± 10.1 % was insufficient for 413 pmol mL^{-1} dye-labelled M13 to reach the threshold for lasing (cyan) and so this spectral data has not been fitted to the same model. Source data are provided as a Source Data file.

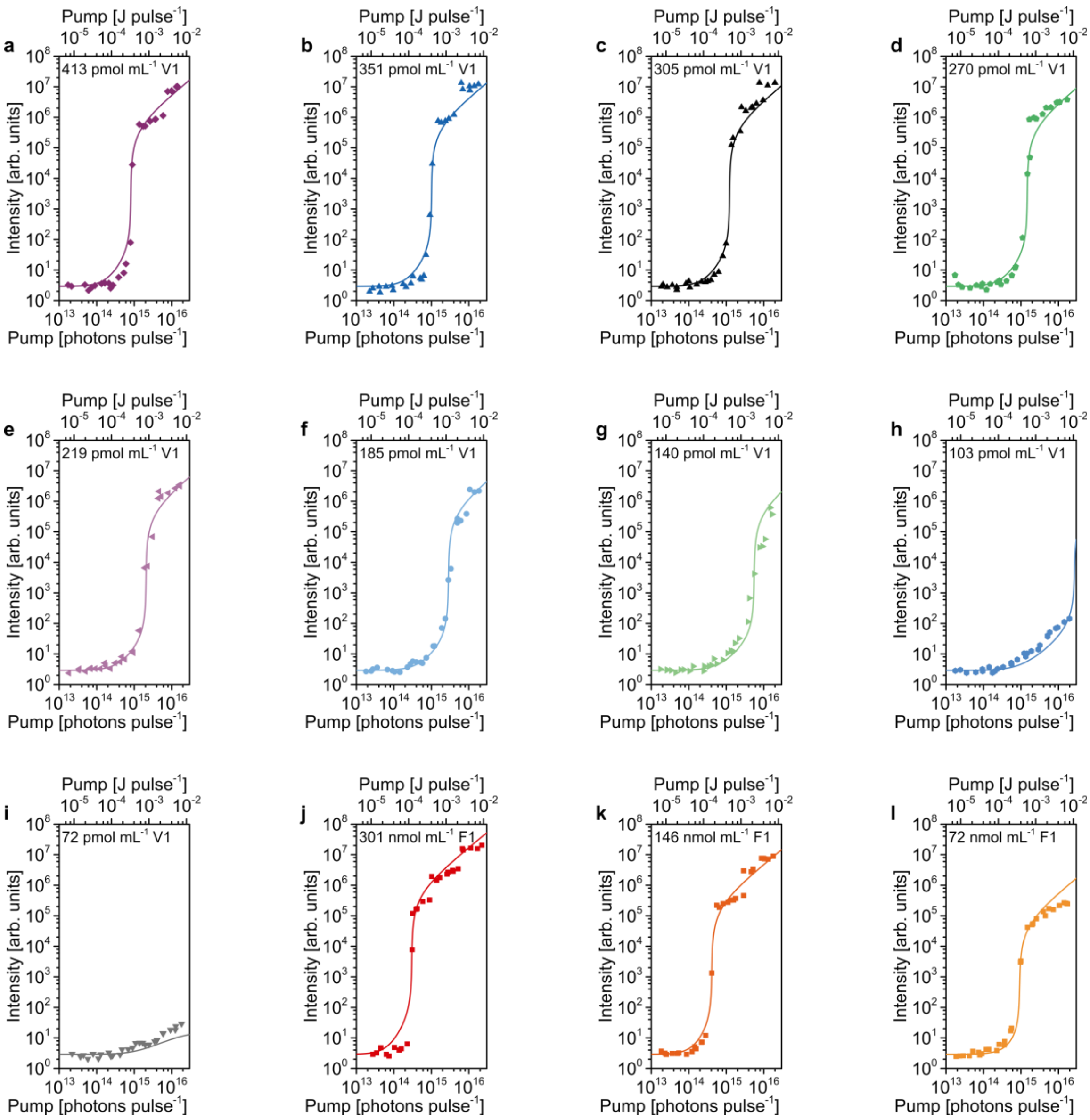

Supplementary Figure 5 | Global fit of the R1 threshold dynamics. The global fit to the theoretical model described in Fig. 2 was performed on, **a-i**, V1 at 9 different probe concentrations and, **j-l**, F1 at 3 different concentrations. The panels show the measured data and fitted curves for each set of data. Source data are provided as a Source Data file.

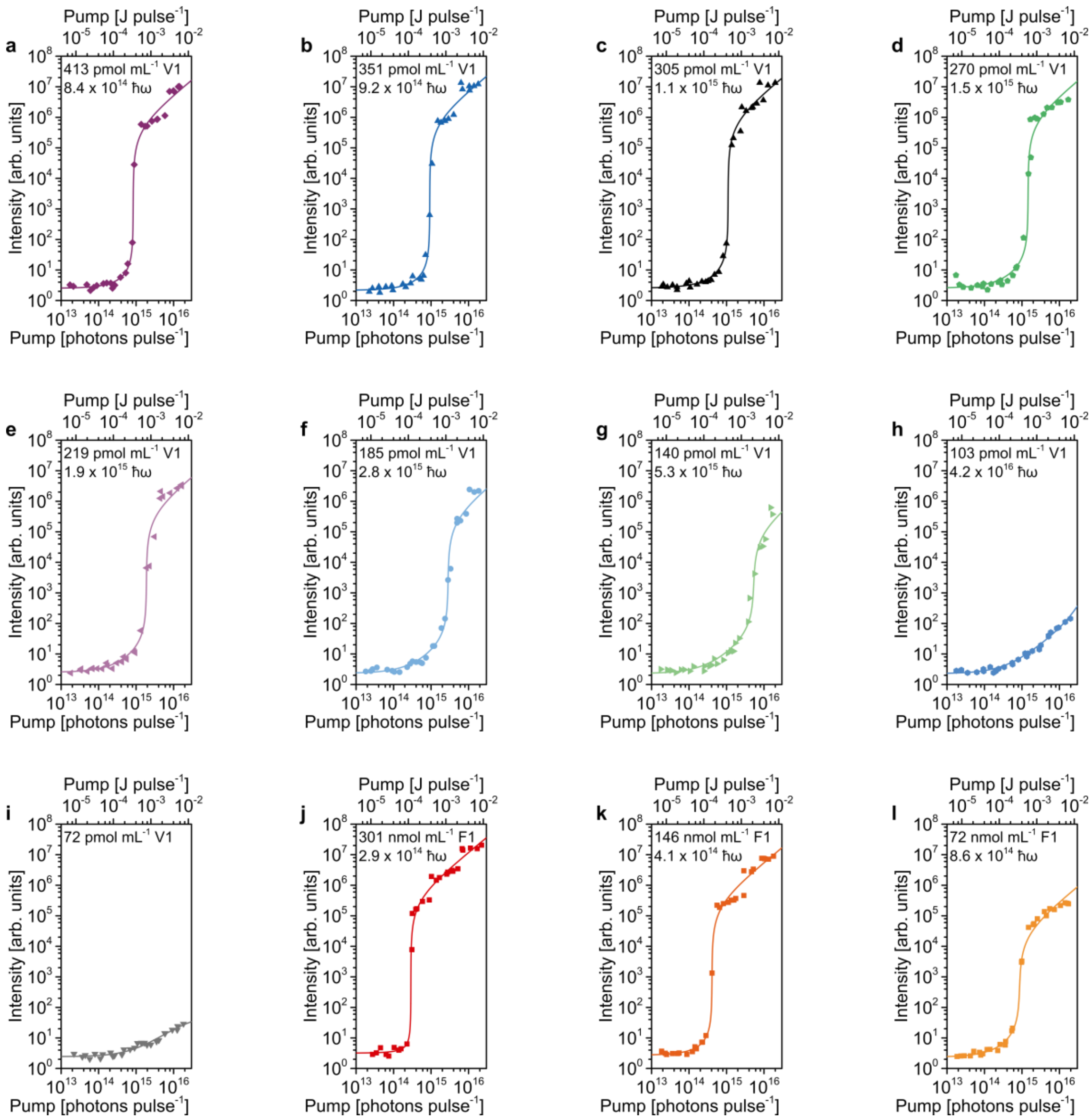

Supplementary Figure 6 | Individual fits of each set of R1 threshold data. Each set of R1 threshold data was fit using an algorithm that did not account for experimental parameters to accurately determine the threshold point (Fig. 3a). The panels show the measured data, fitted curves and threshold points for, **a**-**i**, V1 at 9 different probe concentrations and, **j**-**l**, F1 at 3 different concentrations. The threshold point is displayed in units of ħω at 493 nm, which is equivalent to photons pulse^{-1}. The algorithm returns a negative number for, **i**, 72 pmol mL^{-1} V1, indicating that there was insufficient gain to achieve lasing. From the model fit for 413 pmol mL^{-1} V1, a 2.0-fold increase in the pump energy about the threshold

point results in a 16,000-fold increase in the intensity. Source data are provided as a Source Data file.

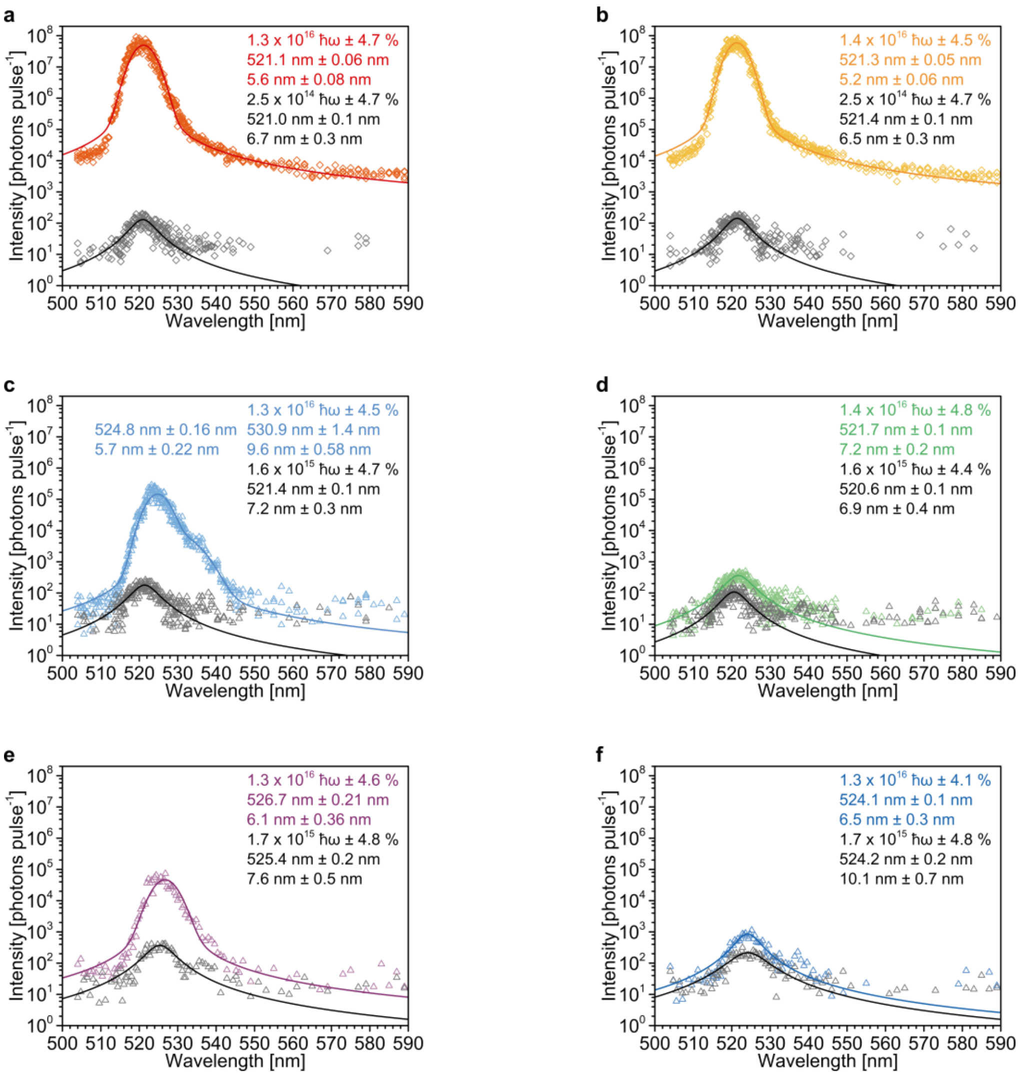

Supplementary Figure 7 | R2 emission spectra. Panels **a**, **b**, **c** and **d** are spectra from the same experiment as described in Fig. 4. The scatter points represent single measurements, unlike in Fig. 4 where the scatter points represent the mean intensity at each wavelength. Panels **e** and **f** are spectra from further experiment 2 in R2. Spectra at higher pump energies are in colour, and spectra at lower pump energies are in grey. For each panel, the legend contains the mean and coefficient of variation of the pump energy and the peak position and FWHM with standard errors for each set of data in colours that match the scatter points and fitted curves. The mean pump energy is displayed in units of ħω at 493 nm, which is equivalent to photons pulse^{-1}. **a**, 13.3 nmol mL^{-1} F2, no cp-mAb. **b**, 13.3 nmol mL^{-1} F2, 9.1 pmol mL^{-1} cp-mAb. **c**, 23 pmol mL^{-1} V2, no cp-mAb. **d**, 22 pmol mL^{-1} V2,

90 fmol mL^{-1} cp-mAb. **e**, 20 pmol mL^{-1} V2, no cp-mAb. **f**, 20 pmol mL^{-1} V2, 4.5 pmol mL^{-1} cp-mAb. Source data are provided as a Source Data file.

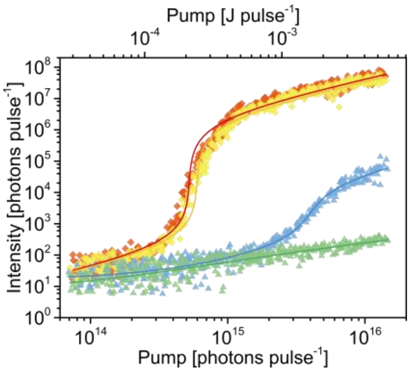

Supplementary Figure 8 | R2 threshold dynamics. Threshold behaviour of V2 and F2 before (cyan, dark orange) and after (light green, yellow) the addition of antibody. The lines represent individual fits of the data to equation (26) in Methods. Source data are provided as a Source Data file.

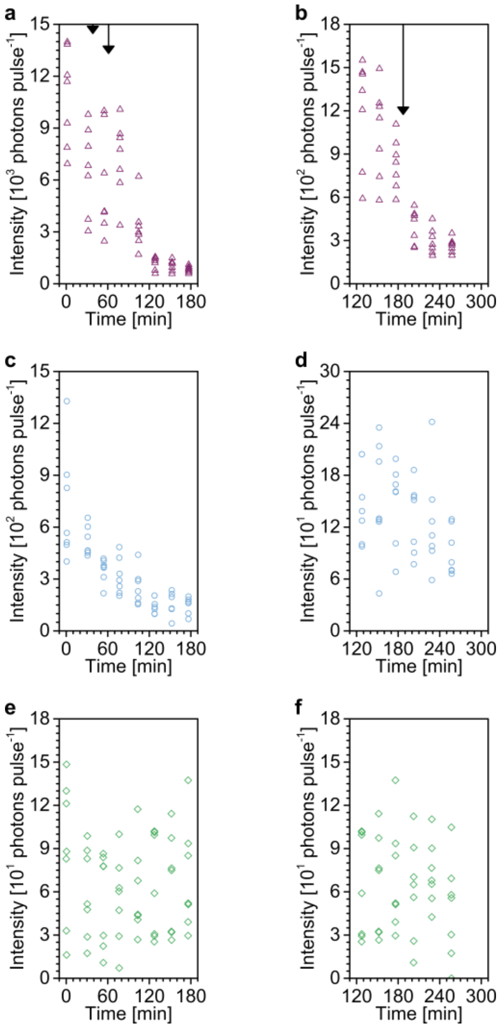

Supplementary Figure 9 | Ligand-binding to lasing detection probes. Time-dependence of the output intensity of the virus laser at, **a**, **b**, 1.3 x 10^{16} photons pulse^{-1} ± 4.7 %, **c**, **d** 4.7 x 10^{15} photons pulse^{-1} ± 5.1 %, and, **e**, **f** 1.6 x 10^{15} photons pulse^{-1} ± 4.4 % on linear scales. The position and relative lengths of the arrows match those in Fig. 4c. Each scatter point represents a single measurement, and the fluctuation in the intensity at each measurement time for each pump energy is partly due to pulse-to-pulse variations in the pump pulse energy. Source data are provided as a Source Data file.

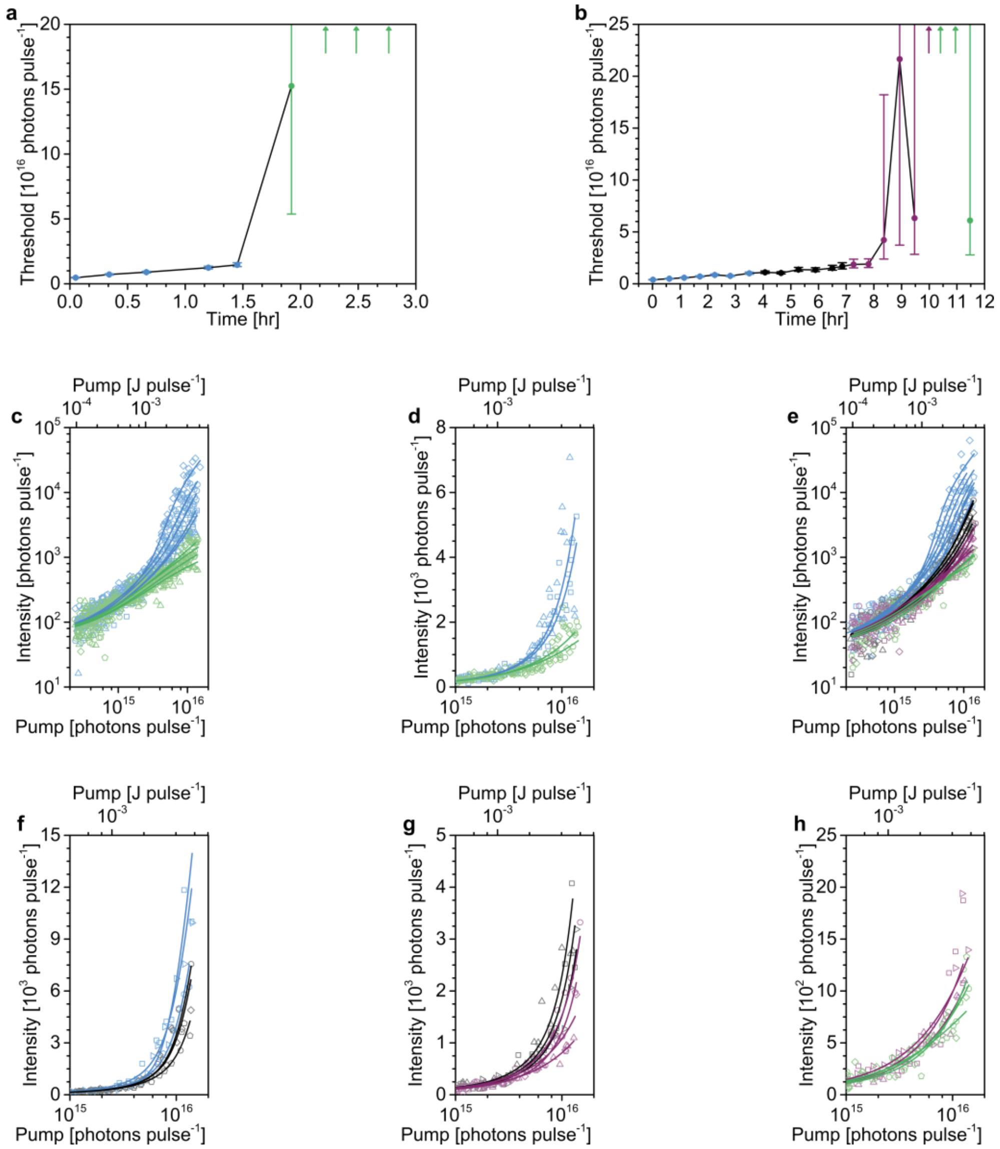

Supplementary Figure 10 | Ligand-binding in further experiments in R2. This figure is an expansion of Fig. 5 with the intensity against pump energy data for various points in the time series. In two further experiments in R2, the threshold behaviour of dye-labelled M13 was monitored as a function of time and the addition of cp-mAb or a non-binding antibody. For each experiment, the data were fit globally using equation (26) with parameters χ_0 and χ_1 shared between each set of threshold measurements. For further experiment 2, 20 pmol mL^{-1} V2 contained no antibody (measured data: cyan; fitted curves: blue), before 4.5 pmol mL^{-1} cp-mAb was added (light green; green). For further experiment 1, 23 pmol mL^{-1} V2 initially contained no antibody (cyan; blue), 91 fmol mL^{-1} mouse IgG2a

isotype control was added (grey; black) and then cp-mAb was added in two steps so that the concentration of cp-mAb was initially 91 fmol mL^{-1} (light purple; purple) and then increased to 1.9 pmol mL^{-1} (light green; green). See Methods for more details. The threshold points derived from the fitted models have been plotted against time for, **a**, further experiment 2 and for, **b**, further experiment 1. Measurements have been represented by an arrow if the fitting implied that the sample could no longer sustain lasing. The error bars represent standard errors and the error bars that extend beyond the upper limit of the y-axis extend to infinity. The measured data and the fitted curves from the global fits are displayed for, **c**-**d**, further experiment 2, and, **e**-**h**, further experiment 1. **c** and **e** show all of the measured data and fitted curves for each experiment and **d**, **f**-**h** show sets of measurements and fitted curves preceding and following the addition of antibody. Photobleaching caused a steady increase in the threshold point as time elapsed in both further experiments, and step-changes in the threshold point appear to coincide with the addition of cp-mAb. Source data are provided as a Source Data file.

Dilution factor	Plaques
10^{0}	Complete clearing of bacterial lawn.
10^{-1}	Complete clearing of bacterial lawn.
10^{-2}	Complete clearing of bacterial lawn.
10^{-3}	Complete clearing of bacterial lawn.
10^{-4}	Complete clearing of bacterial lawn.
10^{-5}	Complete clearing of bacterial lawn.
10^{-6}	Complete clearing of bacterial lawn.
10^{-7}	Complete clearing of bacterial lawn.
10^{-8}	Complete clearing of bacterial lawn.
10^{-9}	Partial clearing of bacterial lawn.
10^{-10}	10
10^{-11}	1
10^{-12}	1

Supplementary Table 1 | Plaque titer assay. Individual plaques were counted manually.

The titer is estimated to be $\frac{10 \times (10 \times 10^{10}) + 1 \times (1 \times 10^{11}) + 1 \times (1 \times 10^{12})}{(10 + 1 + 1) \times 10\ \mu\text{L}} =$ 1.8×10^{13} plaque forming units mL^{-1}, which is ~ 10^{13} plaque forming units mL^{-1}. This confirms that the dye-labelled M13 remained infective because non-infective M13 would not form plaques and therefore yield 0 plaque forming units mL^{-1}.
